# Exploring dental students' knowledge on oral cancer prevention: a cross-sectional study in Moldova, Armenia, and Belarus

**DOI:** 10.1186/s12903-025-05459-8

**Published:** 2025-01-16

**Authors:** Olga Golburean, Diana Uncuta, Gayane Manrikyan, Natalia Shakavets, Izabella Vardanyan, Marina Markaryan, Ferda Özkaya, Daniela-Elena Costea, Tarig Al-Hadi Osman

**Affiliations:** 1 Department of Neuromedicine and Movement Science, Faculty of Medicine and Health Sciences, NTNU-Norwegian University of Science and Technology, Trondheim, Norway; 2 Department of Stomatological Propedeutics “Pavel Godoroja”, Faculty of Stomatology, State University of Medicine and Pharmacy “Nicolae Testemiţanu”, Chisinau, Moldova; 3 Department of Therapeutic Stomatology, Faculty of Stomatology, Yerevan State Medical University, Yerevan, Armenia; 4 Department of Pediatric Dentistry, Faculty of Dentistry, Belarusian State Medical University, Minsk, Belarus; 5 Department of Pediatric Dentistry and Orthodontics, Yerevan State Medical University, Yerevan, Armenia; 6 Freelancer, Bexbach, Germany; 7 Center of Cancer Biomarkers CCBIO, Department of Clinical Medicine, Faculty of Medicine, University of Bergen, Bergen, Norway; 8 Department of Pathology, Haukeland University Hospital, Bergen, Norway; 9 Department of Clinical Medicine, University of Bergen, Bergen, 5020 Norway

**Keywords:** Oral cancer, Dental students, Prevention, Tobacco

## Abstract

**Background:**

Survival rate of patients with oral cancer (OC) remains to be very low despite advancements in therapy and surgical techniques. This is attributed to the fact that most OC cases are discovered at a late stage. Dentists play a vital role in early detection of OC through oral mucosal examination, and in informing the patients about avoidable risk factors of the disease, such as tobacco and excessive alcohol use. This study aimed at evaluating knowledge about OC among dental students in Moldova, Armenia and Belarus; three former soviet countries with high rates of tobacco use.

**Methods:**

This was a cross-sectional, multi-country study based on self-administered questionnaire. Dental students in their clinical years at three dental faculties in Moldova, Armenia and Belarus were invited to participate in the study. Data collection took place during the period May to September 2019. Association between different categorical variables was investigated using Chi-squared test. A knowledge score ranging from 0–14 was constructed from the obtained data. Differences in the knowledge score between different groups of students was investigated using either student`s t-test whenever comparing two groups, or One-way ANOVA with Bonferroni`s correction for three or more groups. The level of significance was set to 0.05 for all statistical tests.

**Results:**

A total of 642 dental students participated in the study. The mean knowledge score was found to be 6.62 ± 2.61, with 45% of the students scoring below the mean. Students from Belarus had the highest score (7.3 ± 2.14) in comparison to Armenia (6.66 ± 2.64) and Moldova (5.66 ± 2.81), as revealed by ANOVA test. However, we observed a significant increase (*p*-value < 0.001, ANOVA) in the students` knowledge score as they proceed through study years from third (6.07 ± 2.61) to fifth year (7.49 ± 2.48). In addition, we found that Students with current or previous cigarette smoking habits had significantly (*p*-value < 0.001, student`s t-test) lower mean knowledge score (5.96 ± 2.82) when compared to students who have never been smokers (6.98 ± 2.42).

**Conclusions:**

The present study reveals notable gaps in OC knowledge and concerning tobacco use behaviors among dental students in Moldova, Belarus, and Armenia. By providing comprehensive education on risk factors and addressing personal habits, dental schools can better prepare future professionals to play a critical role in OC prevention and management.

**Supplementary Information:**

The online version contains supplementary material available at 10.1186/s12903-025-05459-8.

## Background

Oral cancer (OC) is one of the most common cancers in the world with a low survival rate and poor quality of life of survivors that affects millions of people [1–3]. The most common type of OC is oral squamous cell carcinoma (OSCC), which originates from the epithelium of various oral structures such as the lips, mouth floor, buccal mucosa, gingiva, hard palate, and mobile tongue [4, 5].

Despite the progress made in therapy, the rates of occurrence and death caused by OC are still on the rise, particularly in low-income countries [3]. This is primarily due to relatively limited progress towards early detection and diagnostic methods [6]. Thus, OC is often discovered at an advanced stage, despite its location in a readily accessible body region and the presence of easily detectable oral potentially malignant disorders (OPMDs) like leukoplakia and erythroplakia, which can progress into OSCC [6, 7].

Multiple risk factors contribute to the development of oral cancer, including tobacco use, excessive alcohol consumption, poor dietary habits (such as low intake of fruits and vegetables), older age, Human Papilloma Virus (HPV) infection, and familial and genetic predisposition [8–10]. Tobacco consumption is particularly dangerous due to its carcinogenic chemicals, which cause DNA damage and mutations, hindering DNA restoration [11]. Smokers are three times more likely to develop OSCC compared to non-smokers [11, 12]. The risk is further increased when alcohol and tobacco are combined due to their synergistic effects [11, 13].

The prevalence of heavy smokers is notably high in Moldova, Belarus, and Armenia [14–17]. In 2013, the smoking rate among adults in Moldova was 25.3% [15], while Belarus recorded a rate of 25.9% in the same year [16]. Similarly, Armenia reported a smoking rate of 25.4% among adults in 2012 [17]. Moldova and Belarus exhibited high levels of alcohol consumption, with per capita rates of 15.2 L and 11.2 L, respectively, in 2016 [18]. Accordingly, OC represents a significant part of cancer-related deaths in these country, comprising 2,0% of all cancer-related deaths in Moldova [19], 2,3% in Belarus [20], and 0,55% in Armenia in 2022 [21]. In addition, an increase in OC mortality among women have been reported in Moldova and Belarus [22].

According to the World Health Organization, it is possible to prevent 30% to 50% of cancer deaths by reducing key risk factors, implementing effective screening programs, and ensuring early diagnosis and treatment [23]. Furthermore, if the lesions are small and the patient receives adequate treatment, a survival rate of 70% to 90% can be achieved [24, 25]. Unfortunately, the probability of discovering a tumor at advanced-stage is high [1, 26], with only 30% of cancer cases being detected at an early stage [27, 28]. In the case of OC, symptoms may not be apparent in its early stages, which emphasizes the reliance on a clinician's examination findings and a biopsy for diagnosis [29, 30]. Thus, dental practitioners play a vital role in preventing and detecting OC, emphasizing the significance of their knowledge of OC prevention and oral mucosal examination.

Numerous studies have investigated the knowledge on OC among dentists and dental students [31–33]. Interestingly, several studies have identified that few dental professionals discuss/inform their patients about OC risk factors and/or provide counseling to their patients [27, 34–37]. Additionally, several studies have pointed out knowledge and practice gaps among dental students and interns [38–40], emphasizing the urgent need to strengthen the dental curriculum and implement educational programs to promote awareness and knowledge about OC.

While we have previously evaluated dentists' knowledge on OC in Moldova, Belarus, and Armenia [14], to our knowledge there is limited research on dental students' understanding of OC in these countries. Hence, the aim of this study was to assess the knowledge of dental students in the capital cities of Moldova (Chisinau), Belarus (Minsk), and Armenia (Yerevan) regarding OC prevention and oral mucosal examination.

## Methods

### Study design and settings

Belarus has two dental schools: the Belarusian State Medical University in Minsk, and a state dental school in Vitebsk with an enrollment of approximately 220 to 250 students. In Moldova, there is just one dental school, the Moldavian Faculty of Stomatology at the State University of Medicine and Pharmacy “Nicolae Testemiţanu”. In Armenia, the primary dental school is the Yerevan State Medical University, in addition to five smaller cooperative dental schools in Armenia, collectively enrolling around 80 students. This multi-country cross-sectional study utilized a structured, self-administered questionnaire distributed to dental students in Minsk (Belarus), Chișinau (Moldova), and Yerevan (Armenia) who were in their clinical years of study (3rd, 4th, and 5th year). Data collection took place from May 2019 to September 2019. The project was registered at the Norwegian Centre for Research Data (NSD) under Project No: 471282 and 57,451, and also received approval from the ethical committees in Moldova (Comitetului de Etica a Cercetarii, Nicolae Testemitanu, application number 29, date: 20.12.2017), Belarus (Belarusian State Medical University, Protocol number: 10, Date: 20.05.2019), and Armenia (Ethics Committee of Yerevan State Medical University, protocol number: 12–15/2019).

### Data collection

All clinical-year dental students at the Belarusian State Medical University, at the Yerevan State Medical University, and at the Moldavian Faculty of Stomatology, State University of Medicine and Pharmacy “Nicolae Testemiţanu” were invited to participate in the study. In order to have a more homogenous study population, international students in the respective dental schools were not included in the study. Questionnaires were administered in an ordinary classroom at the faculty visited. The students were asked to participate in the study and to sign an informed consent if they were willing to participate (Convenience sampling). The data collector was waiting for the consenting dental students to fill the questionnaire and collected them afterwards. Data from all student that agreed to participate in the study were entered in Excel-file. The questionnaire was anonymous, and participation was voluntary. The participants were provided with both written and verbal information about the study.

### Questionnaire

The closed-ended questionnaire used in the present study consisted of 58 items, divided into six sections. These sections included (1) personal data, (2) oral hygiene, dietary behavior, and utilization of dental services, (3) competency and orientation in preventive care, (4) preventive knowledge, (5) preventive practice for patients and (6) oral mucosal screening and oral cancer prevention (Additional file 1). The questionnaires were developed after reviewing pertinent literature [41–44] and underwent a pilot testing phase involving 10 dental students from each of the three countries. Initially prepared in English, the questionnaire was subsequently translated into the local languages of the respective countries (Romanian, Russian, and Armenian).

The focus of the analysis in this study was on Sect. 6, which pertains to oral mucosal screening and OC prevention. Questions 43–51 from Sect. (6) were used to assess dental students' level of knowledge about OC risk factors, OPMD lesions, most common sites for OC, and clinical properties of early OC lesions. Each correct answer on questions 43–48 was given a score of "1". For question 49, a score of "3" was given for selecting all correct options (floor of the mouth, tongue, rim of tongue), a score of "2" for choosing two options, and a score of "1" for choosing one option. For question 50, a score of "2" was given for selecting leukoplakia and erythroplakia, and a score of "1" for choosing one of them. For question 51, a score of "3" was given for selecting all correct options (small, painless, indurated ulceration; small, painless white area; small, painless red area), a score of "2" for choosing two options, and a score of "1" for choosing one option. The level of knowledge for dental students was determined by the total number of points accumulated, ranging from 0 to 14. The knowledge score was then categorized as 0 = lower knowledge score (0–7) or 1 = higher knowledge score [8–14].

Additionally, three questions regarding dental students' tobacco use from Sect. "Methods", one question about whether dentists serve as role models for their patients and the public from Sect. "Discussion", and one question about whether dental students provide counseling to patients regarding tobacco cessation from Sect. "Conclusions" were also included in the analysis.

### Statistical analysis

All analyses were performed using IBM SPSS Statistics version 29.0 (IBM Corporation, Armonk, NY, USA). The internal consistency of the questionnaire section to be analyzed was investigated using Cronbach test, only questions 43 to 48 were included in this analysis but not the last three questions (49 through 51) because they included multiple choices that are unique for each question. In all analysis, the calculated knowledge score and the variables/questions it is composed of were considered as dependent variables. Descriptive statistics were reported using means and standard deviations (SD) for continuous variables and frequency with percentages for categorical variables. After performing the descriptive statistics, the variables from questions 11, 12, 13, 27, 33, and 34, were transformed into binary variables. For questions 11, 12, and 13, which pertain to cigarette smoking, water-pipe smoking, and snuff or chewing smokeless tobacco respectively, the responses were categorized as "never" = 0, and all other answers “yes” = 1. Variables for question 27 regarding dentists serving as “role models” for their patients and the public, were grouped as agree or strongly agree “yes” = 1, while all other answers were categorized as “no” = 0. for questions 33 and 34, which concern providing counseling to patients regarding tobacco cessation and alcohol cessation, responses indicating "quite often" and "almost always" were grouped as “yes” = 1, and all other answers were categorized as “no” = 0. Bivariate relationships were assessed using chi-square tests. Student t-tests were used to compare means between two groups, while one-way ANOVA with Bonferroni correction was employed to compare means among more than two groups. Multiple linear regression was performed to identify the best model for predicting knowledge score (the dependent variable) considering the following variables (Independent variables): gender, cigarette smoking, country, year of study, tobacco and alcohol counseling, role model, and age group.

## Results

### Characteristics of the study participants

A total of 642 dental students participated in the study. The response rates for dental students in Moldova, Belarus, and Armenia were 51.2% (146/285), 59.7% (187/313), and 55.0% (309/562) respectively. The mean age of the participating students was 21.56 ± 2.16. Notably, the mean age of students from Moldova (23.83 ± 3.04) was significantly higher than that of the other two countries (*p* < 0.001, one-way ANOVA). The majority of participants from all three countries (58.9%) were females (female:male ratio = 1.4:1), and this trend was consistent within each country (Table 1). The distribution of study participants across different study years is also presented in Table 1.

**Table 1:**
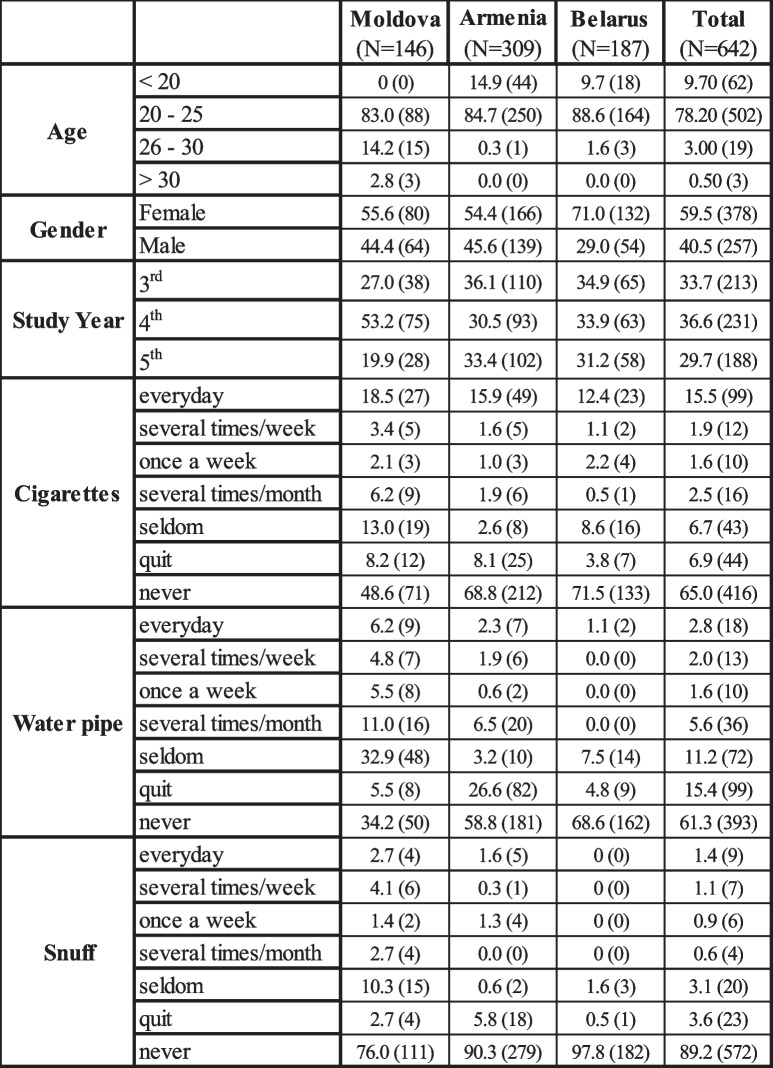
Characteristics of study participants. Data presented as % (n). Some figures are subject to missing data; values may not add up to the total sample

### Use of tobacco among dental students

The data from the study shows that the majority of students (65%) have never been cigarette smokers, while 15.5% reported smoking cigarettes on a daily basis (Table 1). When examining the data from each country individually, Moldova presented with the highest percentage of daily smokers (18.5%), while Belarus had the highest percentage of students who had never been cigarette smokers. A similar trend was observed for the use of water pipes or smokeless tobacco, with Belarus having the highest percentage of students who have never tried it (Table 1).

Furthermore, the use of all types of tobacco investigated in this study was found to be more frequent among male dental students (Table 2). However, in Moldova, the number of female dental students who have never used tobacco was relatively low compared to Armenia and Belarus. More than half of the female students in Moldova (56.3%) have smoked water pipe before, while the majority of females in Armenia and Belarus reported that they have never tried to smoke water pipes (Table 2).

**Table 2:**
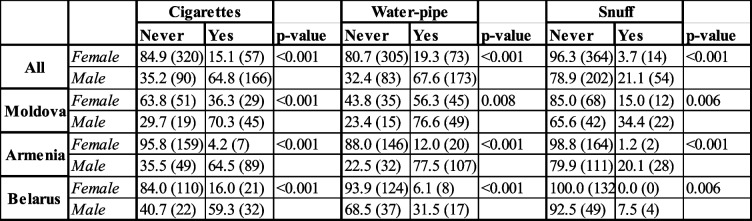
Use of tobacco among dental students and distribution of data by country and gender. Data presented as % (n), and statistical significance of data distribution was investigated by Chi^2^ test. Some figures are subject to missing data; values may not add up to the total sample

In the current study, most of the students answered “agree” or “strongly agree” to the statement that a dentist serves as a role-model to the patients and the public (80.8%). This trend was consistent across different countries, with Moldova (83.6%), Armenia (78.3%), and Belarus (82.9%) displaying similar results. Of note, students who agreed or strongly agreed with this statement were found more likely to have never smoked cigarettes (Additional file 2). While in Moldova and Belarus, there were no statistically significant associations between smoking and giving counseling to patients regarding tobacco or alcohol cessation, in Armenia, however, non-smoker students were significantly more likely to give counseling regarding tobacco cessation than students who were smoking (Chi-2 test, *p* < 0.001) (Additional file 2).

### Assessment of knowledge on OC prevention and early detection

Tobacco and prior OC lesions were identified as the major risk factors by the majority of dental students in all three countries (Table 3). Prior OC lesions scored highest in Belarus, being identified by more than 90% of all clinical year students. Abusive use of alcohol was considered as a risk factor to OC highest by the 5th year students in Belarus and Armenia and by the 3rd year students in Moldova 66.7%, 79.4%, and 55.3% respectively. Older age as a risk factor scored highest among students in Belarus (more than 60%). Only 34.7% of the 4th year students in Moldova and 44.5% of the 3rd year students in Armenia reported older age as a risk factor.

**Table 3:**
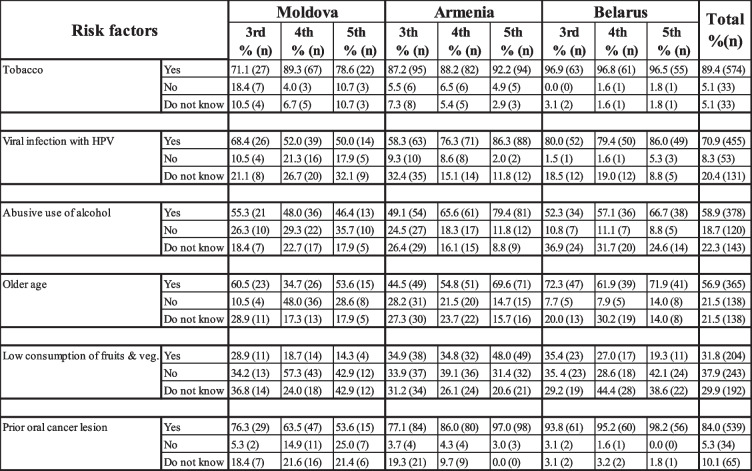
Dental students’ responses about OC risk factors according to the year of study. Data presented as percentages (number)

Most dental students did not consider rim of the tongue as common sites for OC. Floor of the mouth was listed highest by the 5th year students in Moldova (42.9%) and Belarus (39.7%) and by the 3rd year students in Armenia (43.6%). When asked about lesions that were most likely to be OPMDs, leukoplakia was identified by more than 80.0% of all clinical year students in Belarus. In Moldova and Armenia, leukoplakia was mentioned by fewer students compared to Belarus (Table 4). Erythroplakia was mentioned most frequently by 5th year students in Belarus (58.6%) and lowest by 3rd year students in Moldova (5.3%). Slightly more than 40% of 3rd year dental students in Moldova and Armenia reported that they do not know which are the lesions with malignant potential and what are the clinical properties of an early OC lesion (Table 4). Regarding clinical properties of an early cancer lesion, small, painless white area and small painless, indurate ulceration scored highest in all three countries (Table 4).

**Table 4:**
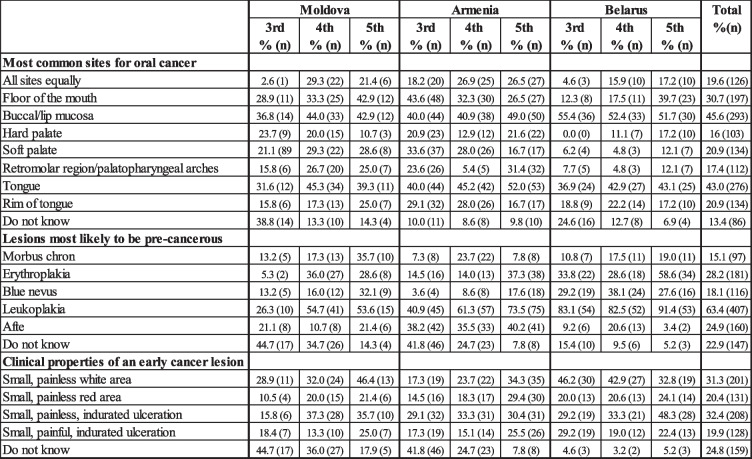
Dental students’ responses about OPMD lesions, risk sites and clinical properties of early OC lesions. Data presented as percentages

### OC Knowledge Score

Investigating internal consistency of the questions from 43 to 48 yielded a Cronach alpha value of 0.613 which indicated a good reliability of this part of the questionnaire. The mean knowledge score of dental students from the three countries together was found to be 6.62 ± 2.61 (Total score is 14). The minimum score was 0 and the maximum was 13 while the mode was found to be 7 scored by only 16% of the study participants. Overall, the study revealed that dental students from all three countries had a relatively low level of knowledge regarding oral cancer risk factors and clinical presentation, with 45% of students scoring below the mean. Female students (Table 5) showed significantly higher mean score (6.92 ± 2.46) when compared to males (6.12 ± 2.78) as revealed by student t-test (*p*-value < 0.001).

**Table 5:**
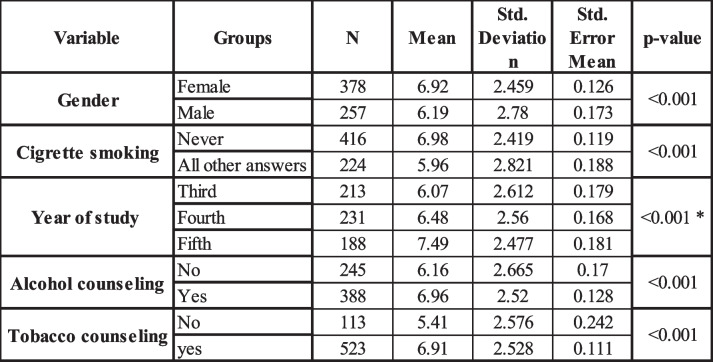
OC knowledge score by gender; cigarette smoking; year of study; Alcohol counseling; and tobacco counseling. Statistical significance of the mean difference between groups was investigated by student t-test except for year of study where ANOVA test with Bonferonni correction was used. * No statistically significant difference was observed between mean scores of third and fourth year students; but score for 5^th^ year students wassignificantly larger than both third and fourth years

Additionally, students who had never smoked cigarettes obtained a significantly higher score (6.98 ± 2.419) compared to students who were current or former smokers (5.96 ± 2.82) as shown by student t-test (*p*-value < 0.001) (Table 5). Furthermore, students in their 5th year of study achieved a higher score (7.49 ± 2.48) than those in their 4th (6.48 ± 2.56) or 3rd (6.07 ± 2.61) year of study, as determined by ANOVA (*p*-value < 0.001). Moreover, students who reported providing counseling to their patients regarding tobacco and alcohol use (quite often, almost always) had higher knowledge scores (tobacco counseling 6.91 ± 2.53, alcohol counseling 6.96 ± 2.52) compared to those who never or seldom offered such counseling to patients (tobacco 5.41 ± 2.58, alcohol 6.16 ± 2.67), as shown by t-test (*p*-value < 0.001 for both) (Table 6). When comparing the data from the three countries, a statistically significant difference (ANOVA, *p*-value < 0.001) in the mean knowledge score was observed between Moldova (5.66 ± 2.81), Armenia (6.66 ± 2.64), and Belarus (7.3 ± 2.14). Furthermore, through multiple linear regression analysis, the key variables for predicting the knowledge score have been identified to be country, year of study, offering alcohol and tobacco counseling, and cigarette smoking (Additional file 3).

**Table 6:**
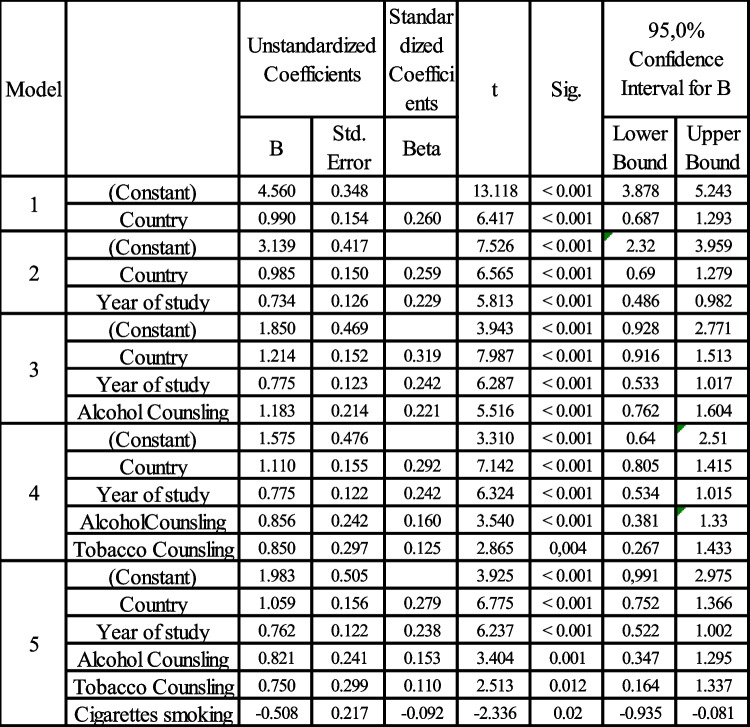
Multiple linear regression analysis reveals that country of the student, year of study, cigarette smoking and answers to the question about offering tobacco and alcohol counsling together comprise the best model for prediction of change in the OC knowledge score

## Discussion

This multi-country study provides a comprehensive examination of the knowledge of dental students in Moldova, Belarus, and Armenia regarding OC prevention and early detection. Our study shows that although the vast majority of dental students identified tobacco use and prior OC lesions as risk factors for OC (89.4% and 84.0% respectively), awareness of other important factors such as excessive alcohol consumption (58.9%), and older age (56.9%) was insufficient. This deficiency in OC knowledge is consistent with similar cross-sectional studies performed among dental students in Malaysia [45], Türkiye [29], Palestine [38], Yemen [39], and Saudi Arabia [40]. The lack of awareness in our study, particularly regarding the impact of alcohol, is concerning given the established synergistic effect of tobacco and alcohol in increasing oral cancer risk [13]. These gaps underscore the need for comprehensive education on all oral cancer risk factors within dental training programs.

The ability to identify OPMDs and early signs of OC is critical for timely intervention [46]. The present study found that while leukoplakia was identified as an OPMD by the majority of the dental students (63.4%), awareness of erythroplakia was notably lower. Moreover, a substantial proportion of students were uncertain about the clinical properties of early OC lesions, with 24.8% indicating that they "do not know." These percentages are alarming, given that early detection of OC significantly improves prognosis and survival rates [24, 25]. During their clinical years, dental students must be thoroughly trained to recognize the signs and symptoms of early-stage OC, as well as to distinguish OPMDs. This competency allows them to make informed decisions about when to perform biopsies or refer patients to specialist clinics [1]. Training dental students in early detection not only enhances patient outcomes but also has economic implications. By identifying OC early, dentists can contribute to reducing the overall financial burden on healthcare systems [47].

This study reveals a concerning gap in OC knowledge among the surveyed students, with a low mean knowledge score (6.62 out of 14) across all the three countries. Regression analysis performed in the present study indicated that the OC knowledge score among dental students was influenced by factors such as study year, patient counseling practices, gender, and country. Encouraging is the finding that the score shows an upward trend with each year of study, indicating a strengthening of knowledge as students progress through their studies. The increasing knowledge score with each academic year is expected, as it reflects the cumulative effect of education and clinical exposure on students’ understanding of OC [38, 39]. Similarly, students who engage in patient counseling regarding tobacco and alcohol use are likely to have better knowledge of OC, as counseling requires a solid understanding of OC risk factors and preventive measures [48]. The finding that female dental students exhibited higher knowledge scores (6.92) compared to their male counterparts (6.12) also aligns with previous research [49–51]. Indeed, studies have shown that female dental students often score higher in oral health knowledge assessments compared to male students, suggesting that female students are more engaged in their dental studies than their male counterparts [49–51]. Additionally, the gender disparity in knowledge observed in this study might be influenced by the demographic composition of the student sample, which included 59% female participants. As dentistry has increasingly become a female-dominated profession, similar demographic trends have been reported in other cross-sectional studies of dental students in countries such as Malaysia [33], Yemen [39], Estonia [52], and Palestine [38].

The overall knowledge score for dental students observed in the current study is also lower than the overall knowledge score we detected in our previous study among dentists in the same countries (mean ± SD knowledge score for all countries combined was 7.5 ± 2.7) [14], suggesting that the postgraduate studies and practice as a dentist plays an important role for the knowledge on OC and OPMDs in these countries. Nevertheless, the findings of this study suggest that dental education programs in Moldova, Armenia, and Belarus need to undergo a thorough review and revision to ensure that they are effectively covering all aspects of OC. Enhancing the curriculum could involve more hands-on training, case studies, and interdisciplinary learning opportunities [33, 39, 53].

The high prevalence of tobacco use among dental students in this study is noteworthy with 15.5% of the students reported daily cigarette smoking, with male students significantly more likely to smoke compared to female students. Specifically, in Moldova, higher percentage of students reported daily smoking as compared to students from the other two countries. This higher prevalence could be attributed to the fact that students in Moldova are generally older, as older individuals may have had more time to develop or maintain smoking habits. These findings resonate with global patterns of tobacco use among dental students [54–56]. Among dental students in Jordan, India, and Israel, the prevalence of smoking was 17%, with smoking more prevalent in male students than female [54–56]. It is noteworthy here the high rates of tobacco use, including water-pipe smoking, by female dental students in Moldova as compared to the other two countries. These observations might be explained by cultural differences between the three countries included in this study which justifies more in-depth future qualitative research into this phenomenon, preferably in collaboration with researchers with academic background in social science. The high rates of tobacco use among dental students are concerning, especially given their role as health promoters. Dentists who smoke may be less inclined to counsel patients on tobacco cessation, as evidenced by our study's finding that non-smoker students were more proactive in providing such counseling than students who were smoking, particularly in Armenia. In addition, non-smoker dental students in the present study achieved a significantly higher knowledge score (6.98) compared to smokers (5.96). It is thus clear that personal smoking habits can influence dental students' attitudes towards tobacco cessation counseling and their retention of knowledge related to oral cancer prevention [57]. Consequently, incorporating tobacco cessation programs within dental schools and fostering a culture that discourages tobacco use can enhance the effectiveness of future dentists in promoting cessation among patients.

The present study has some limitations that need to be acknowledged. There was a 5 years gap between data collection and reporting of the data, and the estimates reported here might have changed during the time period it took to analyze and publish the data. In addition, our study had an overall response rate of 55.3%. While this response rate is higher than in other previous studies [39, 40], it may still introduce response bias and limit the generalizability of the findings. Furthermore, it is possible that students who felt more confident in their knowledge of oral cancer were more inclined to participate in the study. In addition, the study’s cross-sectional nature limits the ability to draw causal inferences, for example between insufficient OC knowledge and tobacco use history of the students. Another study limitation is the reliance on self-reported data for smoking behaviors and knowledge, which may introduce social desirability and recall biases. Nevertheless, our study is strengthened by the use of a rigorously developed and pilot-tested questionnaire. Another key strength of this study is its multi-country design, which enhances its external validity and allows for the assessment of dental students' knowledge and behaviors across different cultural and educational contexts. The inclusion of dental students from multiple academic years also provides insight into how knowledge and attitudes evolve as they progress through their dental education.

Our future directions on this topic include performing qualitative study among dental students in the three countries with the aim to explore reasons of the observed insufficient knowledge about OC, and to clarify possible causal associations between OC knowledge level and tobacco use. We have also collected information about oral health behaviors of dental students by using the same questionnaire (Additional file 1), combining these data with the variables included in the current study can facilitate investigating potential association between oral health behaviors of dental students and their motivation to learn about OC prevention and its risk factors. Such analysis might reveal important trends in oral health behaviors of dental students and the effect this might have on fulfilling their role as future dentists in OC prevention.

## Conclusions

The present study reveals notable gaps in OC knowledge and concerning tobacco use behaviors among dental students in Moldova, Belarus, and Armenia. To bridge these gaps, dental education programs should undergo regular revisions to incorporate the latest advancements in OC prevention and early detection. Implementing interactive and engaging curriculum updates—such as hands-on training, case studies, and simulations—can greatly enhance students' understanding and practical skills. By providing comprehensive education on risk factors and addressing personal habits, dental schools can better prepare future professionals to play a critical role in oral cancer prevention and management.

## Supplementary Information

Additional file 1.

Additional file 2.

## Data Availability

The dataset generated and analyzed during the current study is available from the corresponding author upon reasonable request.

## Abbreviations

OC: Oral Cancer
OSCC: Oral Squamous Cell Carcinoma
OPMD: Oral Potentially Malignant Disorders
NSD: Norwegian Center for Research Data
SD: Standard Deviation
ANOVA: Analysis of Variance
Chi-2: Chi-squared test
