# Supplementary material for: Exploring dental students' knowledge on oral cancer prevention: a cross-sectional study in Moldova, Armenia, and Belarus
Source: BMC Oral Health. 2025 Jan 16;25:81. doi: 10.1186/s12903-025-05459-8 (PMC11740607; doi:10.1186/s12903-025-05459-8)
Supplement: Supplementary file 1 — Additional file 1. [file 12903_2025_5459_MOESM1_ESM.docx]

**Additional File – 1**

**Questionnaire**

***(Dental students)***

**No:……….**

**Personal data**

1. Age

……………………….

1. Gender
   1. Female
   2. Male
2. In which year are you studying?
   1. Third
   2. Fourth
   3. Fifth

**Oral hygiene and dietary behavior and utilization of dental services**

1. How often do you usually brush your teeth?

- 1. Never
  2. Less than once a week
  3. Once a week
  4. More than once a week
  5. Once a day
  6. Twice a day or more

1. How often do you clean with *dental floss / interdental brush* between teeth?
   1. Never
   2. Less than once a week
   3. Once a week
   4. More than once a week
   5. Once a day
   6. Twice a day or more
2. Do you use toothpaste-containing fluoride while brushing?
   1. Not at all
   2. Seldom
   3. Quite often
   4. Always or almost always
3. How often do you eat sugar-containing snacks between your main meals?
   1. About three times a day or more
   2. About twice a day
   3. About once a day
   4. Occasionally
   5. Rarely or never between meals
4. How often do you visit a dentist?
   1. More seldom than twice a year
   2. Every second year
   3. Once a year
   4. Twice or more yearly
5. What was the reason for your last dental visit?
   1. Acute/pain driven
   2. Regular check-up
   3. Other, please specify

……………………………………………………………………………………….

1. How would you describe the condition of your mouth and teeth?
   1. Good
   2. Quite good
   3. Average
   4. Quite bad
   5. Bad
   6. Do not know
2. How often do you smoke cigarettes/pipe?
   1. Every day
   2. Several times a week
   3. Once a week
   4. Several times a month
   5. Seldom
   6. Smoked but quit
   7. Never smoked
3. How often do you smoke water-pipe?
   1. Every day
   2. Several times a week
   3. Once a week
   4. Several times a month
   5. Seldom
   6. Smoked but quit
   7. Never smoked
4. How often do you use snuff or chewing smokeless tobacco?
   1. Every day
   2. Several times a week
   3. Once a week
   4. Several times a month
   5. Seldom
   6. I used it before but I quitted
   7. I never used it

**Competency and orientation in preventive care**

1. I feel competent to provide clinical care to my patients
   1. Strongly disagree
   2. Disagree
   3. Do not know
   4. Agree
   5. Strongly agree
2. I feel competent to perform preventive activities to my patients
   1. Strongly disagree
   2. Disagree
   3. Do not know
   4. Agree
   5. Strongly agree
3. I feel competent to give oral health education to my patients
   1. Strongly disagree
   2. Disagree
   3. Do not know
   4. Agree
   5. Strongly agree
4. Theoretical and preclinical training on preventive dentistry in dental undergraduate education should be increased.
   1. Strongly disagree
   2. Disagree
   3. Do not know
   4. Agree
   5. Strongly agree
5. Clinical practice of preventive dentistry in dental undergraduate education should be increased.
   1. Strongly disagree
   2. Disagree
   3. Do not know
   4. Agree
   5. Strongly agree

**Preventive knowledge**

1. Brushing teeth with fluoride toothpaste prevents tooth decay.
   1. Strongly disagree
   2. Disagree
   3. Do not know
   4. Agree
   5. Strongly agree
2. Fluoridation of drinking water is an effective, safe, and efficient way to prevent dental caries.
   1. Strongly disagree
   2. Disagree
   3. Do not know
   4. Agree
   5. Strongly agree
3. It is beneficial to recommend fluoride tablets and/or topical fluorides for children in areas without a fluoridated water supply.
   1. Strongly disagree
   2. Disagree
   3. Do not know
   4. Agree
   5. Strongly agree
4. The frequency of sugar-consumption has a greater role than the total amount of sugar consumed in causing caries.
   1. Strongly disagree
   2. Disagree
   3. Do not know
   4. Agree
   5. Strongly agree
5. Sugar-free chewing gum has a positive effect on oral health.
   1. Strongly disagree
   2. Disagree
   3. Do not know
   4. Agree
   5. Strongly agree
6. Sealant is effective in prevention of pit and fissure caries in molars.
7. Strongly disagree
8. Disagree
9. Do not know
10. Agree
11. Strongly agree
12. It is beneficial to visit a dentist for oral health.
    1. Strongly disagree
    2. Disagree
    3. Do not know
    4. Agree
    5. Strongly agree
13. Regular brushing helps to prevent gum problems.
14. Strongly disagree
15. Disagree
16. Do not know
17. Agree
18. Strongly agree
19. Dentists serve as “role models” for their patients and the public.
    1. Strongly disagree
    2. Disagree
    3. Do not know
    4. Agree
    5. Strongly agree
20. Patients’ chances of performing oral health promoting behaviors increase if health professional advise them.
    1. Strongly disagree
    2. Disagree
    3. Do not know
    4. Agree
    5. Strongly agree

**Preventive practice for patients**

1. Do you recommend fluoride toothpaste to your patients?
   1. Not at all
   2. Seldom
   3. Quite often
   4. Always or almost always
2. Do you give oral hygiene instructions to your patients?
3. Not at all
4. Seldom
5. Quite often
6. Always or almost always
7. Do you give nutrition and diet counselling to your patients?
   1. Not at all
   2. Seldom
   3. Quite often
   4. Always or almost always
8. Do you use topical application of fluoride for your patients?
   1. Not at all
   2. Seldom
   3. Quite often
   4. Always or almost always
9. Do you give counselling to your patients regarding tobacco cessation?
   1. Not at all
   2. Seldom
   3. Quite often
   4. Always or almost always
10. Do you give counselling to your patients regarding excessive alcohol cessation?
    1. Not at all
    2. Seldom
    3. Quite often
    4. Always or almost always
11. Do you recommend regular check-ups to your patients?
12. Not at all
13. Seldom
14. Quite often
15. Always or almost always

**Oral mucosal screening and oral cancer prevention**

1. Do you check all new patients for oral mucosal (buccal, lip, floor of the mouth, tongue, base of tongue, palate, retromolar area and palatopharyngeal arches) lesions?
   1. Yes
   2. No
   3. Do not remember
2. Do you check all recall patients for oral mucosal lesions?
   1. Yes
   2. No
   3. Do not remember
3. Have you ever detected a suspicious lesion for oral cancer?
   1. Yes
   2. No
   3. Do not remember
4. Have you ever performed biopsy of oral mucosa?
   1. Yes
   2. No
   3. Do not remember
5. Checking oral mucosa should occur for all new patients.
   1. Strongly disagree
   2. Disagree
   3. Do not know
   4. Agree
   5. Strongly agree
6. Checking oral mucosa should occur for all recall patients.
   1. Strongly disagree
   2. Disagree
   3. Do not know
   4. Agree
   5. Strongly agree
7. Checking oral mucosa should be targeted to those at high risk of developing oral cancer.
   1. Strongly disagree
   2. Disagree
   3. Do not know
   4. Agree
   5. Strongly agree
8. Do you consider use of tobacco as a risk factor for oral cancer?

- 1. Yes
  2. No
  3. Do not know

1. Do you consider viral infection with HPV as a risk factor for oral cancer?
   1. Yes
   2. No
   3. Do not know
2. Do you consider abusive use of alcohol as a risk factor for oral cancer?
   1. Yes
   2. No
   3. Do not know
3. Do you consider older age as a risk factor for oral cancer?
   1. Yes
   2. No
   3. Do not know
4. Do you consider low consumption of fruits and vegetables as a risk factor for oral cancer?
   1. Yes
   2. No
   3. Do not know
5. Do you consider prior oral cancer lesion as a risk factor for oral cancer?
   1. Yes
   2. No
   3. Do not know
6. Do you consider the most common sites for oral cancer to be *(Tick those that apply)*
   1. all sites equally
   2. floor of the mouth
   3. buccal/lip mucosa
   4. hard palate
   5. soft palate
   6. retromolar region/palatopharyngeal arches
   7. tongue
   8. rim (sides) of tongue
   9. Do not know
7. The two lesions most likely to be pre-cancerous are (*You should tick 2 answers)*
   1. Morbus Chron
   2. Erythroplakia
   3. Blue nevus
   4. Leukoplakia
   5. Afte
   6. Do not know
8. Most common clinical properties of an early cancer lesion *(Tick those that apply)*
   1. small, painless white area
   2. small, painless red area
   3. small, painless, indurated ulceration
   4. small, painfull, indurated ulceration
   5. Do not know
9. Do you ask your patients about current/previous use of tobacco?
   1. Yes
   2. No
   3. Do not remember
10. Do you ask your patients about current/previous use of alcohol?
    1. Yes
    2. No
    3. Do not remember
11. Do you ask your patients about family history of cancer?
    1. Yes
    2. No
    3. Do not remember
12. You in current role as a dental student can influence a patient to reduce/quit smoking or drinking alcohol.
    1. Strongly disagree
    2. Disagree
    3. Do not know
    4. Agree
    5. Strongly agree
13. You in current role as a dental student should provide smoking or alcohol cessation advice.
    1. Strongly disagree
    2. Disagree
    3. Do not know
    4. Agree
    5. Strongly agree
14. It is the role of dentists to perform oral mucosal screening.
    1. Strongly disagree
    2. Disagree
    3. Do not know
    4. Agree
    5. Strongly agree
15. It is the role of medical doctors/family doctor/specialists to perform oral mucosal screening.
    1. Strongly disagree
    2. Disagree
    3. Do not know
    4. Agree
    5. Strongly agree

***THANK YOU!***
