# Supplementary material for: Exploring dental students' knowledge on oral cancer prevention: a cross-sectional study in Moldova, Armenia, and Belarus
Source: BMC Oral Health. 2025 Jan 16;25:81. doi: 10.1186/s12903-025-05459-8 (PMC11740607; doi:10.1186/s12903-025-05459-8)
Supplement: Supplementary file 2 — Additional file 2. [file 12903_2025_5459_MOESM2_ESM.docx]

**Additional file 2.** Distribution of the students` answers regarding the questions about “Dentist as a role model to patients” and “offering tobacco and alcohol counseling to patients” according to the students` smoking history. Data presented as % (n) and p-values were obtained using Chi^2^ test.

| Country | Cigarette smoking | Dentist is a role model % (n) | | Chi-2 | Tobacco Counsling % (n) | | Chi-2 | Alcohol Counsling % (n) | | Chi-2 |
| --- | --- | --- | --- | --- | --- | --- | --- | --- | --- | --- |
|  |  | no | yes |  | no | yes |  | no | yes |  |
| Moldova | Never | 25.0 (6) | 53.3 (65) | 0.01 | 42.5 (17) | 51.0 (53) | 0,235 | 45.7 (21) | 48.5 (47) | 0.447 |
|  | Yes | 75.0 (18) | 46.7 (122) |  | 57.5 (23) | 49.0 (51) |  | 54.3 (25) | 51.5 (50) |  |
|  | Total | 100 (24) | 100 (187) |  | 100 (40) | 100 (104) |  | 100 (46) | 100 (97) |  |
| Armenia | Never | 56.7 (38) | 72.2 (174) | 0.013 | 32.4 (12) | 74.0 (199) | < 0,001 | 49.4 (39) | 75.2 (170) | <0.001 |
|  | Yes | 43.3 (29) | 27.8 (67) |  | 67.6 (25) | 26.0 (70) |  | 50.6 (40) | 24.8 (56) |  |
|  | Total | 100 (67) | 100 (241) |  | 100 (37) | 100 (269) |  | 100 (79) | 100 (226) |  |
| Belarus | Never | 48.8 (15) | 76.1 (118) | 0.003 | 64.7 (22) | 73.3 (110) | 0,211 | 71.2 (34) | 73.8 (48) | 0.419 |
|  | Yes | 51.6 (16) | 23.9 (37) |  | 35.3 (12) | 26.7 (40) |  | 28.8 (34) | 26.2 (17) |  |
|  | Total | 100 (31) | 100 (155) |  | 100 (34) | 100 (150) |  | 100 (118) | 100 (65) |  |
